# The Special Examination of Mental Defectives

**Published:** 1913-11-22

**Authors:** 


### THE SCHOOL DOCTOR'S DIFFICULTIES.

### II.—The Special Examination of Mental Defectives.

Presuming that the school doctor has eliminated all possible physical defects, through the routine examination recommended in the last article, he can test the intelligence of the children by a special examination. Ordinarily, where the work devolves upon the special group of school doctors who are in charge of the physically and mentally defective children attending the special schools, all that is required from the school doctor is to state that, in his opinion, the children are of such a low degree of mental development that it is advisable for them to be specially examined. This is what is meant by "referring children for the statutory examination for the M. D. schools." But where the work devolves upon the school doctor himself the examination means a patient and often very laborious undertaking.

The special examination has for its object the thorough testing of the child's intelligence and the comparison of the results found with what may be taken as the average normal intelligence of a child of the same age and the same social standing. Considerable experience and skill are needed for this test. In the first place the examiner has to win the confidence of the examinee. Secondly, he has to consider very carefully such information with regard to the child's mental powers, school work, moral and intellectual capacity, as may be furnished by the class teacher. It is therefore well that the class teacher, who presumably knows the child fairly intimately, should attend the examination and assist the doctor. Before the examination the teacher compiles a form in which particulars are given of the child's habits, work, social conditions, life history, and ancestry. Attention to trifling points is vitally necessary, but too much consideration must not be given to them. While, for instance, it is well to know that a certain child's brother or sister is attending a special school for mentally defective children, this fact must not be taken as direct evidence of the child's own mental defect. The routine tests of intelligence are fairly simple; it is the interpretation of the findings that demands special care.

The most popular method of investigating the child's mentality is that which is associated with the names of Binet and Simon. In this method the intellectual level of the child is estimated according to the child's response to certain definite tests which have been drawn up as the result of a long and carefully conducted examination of hundreds of normal children. A full description of the manner in which the test is applied and interesting suggestions with regard to the examination were given by Binet in his paper in "L'Année Psychologique" for 1905, and an interesting paper, dealing with the results of special tests upon defective children in the Summerton Special School, was contributed by Dr. Kate Fraser to the July number of "School Hygiene."

The Binet-Simon tests are arranged in several groups. In the first the child's powers of description are tested by showing him simple pictures of which he is afterwards asked to give details. A three-year-old child ought to be able to recognise simple objects in a picture; older children should be able to tell the nature and character of the objects noted, and, in the senior groups, to be able to give reasons for the answers. In the second group are the tests for the recognition and definition of common objects so as to show the child's power of expression. A seven-year-old child, for instance, should be able to define the use of a common object, such as a pencil or table fork. This test, in Dr. Fraser's opinion, is not satisfactory, "as it depends to a large extent on attention and concentration as well as on memory." A third test is the judgment of length by the comparison of lines of unequal length; a fourth, somewhat similar, the comparison of weights; at five years the normal child is able to compare fairly well two weights of different values; at nine years he can arrange five different weights. In mentally defective children the sense of weight is generally feeble.

Other tests are comparison of two objects from memory, which is usually considered a valuable test; tracing resemblances between two objects; framing simple sentences; recognising obvious defects in pictures; carrying out a simple order; matching two or more colours; answering simple questions, and counting tests. Various modifications of these are employed, but they all have a common objective—namely, to ascertain whether or not the child responds in a manner which enables him to be placed in one or other of the age groups into which, in accordance with these tests, children who are not mentally defective can be ranged. A child of seven may be found to be intellectually on a level with a normal child of four; this is evidence of retardation of intellect and may be due to some other cause—for example, bad teaching—than mental deficiency. A child who responds to the Binet-Simon tests for his age is not mentally defective in the ordinary sense of the term, although his moral and intellectual development may be such that he is not fitted for an ordinary school. The moral imbecile, for example, may respond very well to the tests, and cannot therefore be grouped under the mental defectives. Such children are a source of great worry and perplexity to head masters and mistresses, but in present circumstances it is difficult to see what can be done with them. Their proper place is really in a home or in an industrial school, but the school doctor has no power to place them there. In the present state of the law a child can only be sent to an industrial school by a magistrate; in other words, the child must commit some action which makes him amenable to the law before he can enter what is after all the best school for him. This is undoubtedly a defect under the present system, but until the law is altered it is impossible to deal with them.

